# Social Policies and Theories on Quality of Life under COVID-19: In Search of the Missing Links

**DOI:** 10.1007/s11482-023-10147-2

**Published:** 2023-02-24

**Authors:** Daniel T. L. Shek, Janet T. Y. Leung, Lindan Tan

**Affiliations:** Department of Applied Social Sciences, The Hong Kong Polytechnic University, Hung Hom, Hong Kong

## Abstract

COVID-19 has generated many negative impacts on the family, including impaired psychological well-being of family members (parents and children) and family processes (such as parenting and family functioning). Regarding social policies to support families under the pandemic, there are several missing links. First, COVID-19 related policies mainly focus on physical well-being with a relatively weaker emphasis on psychological well-being. Second, with social policies primarily aiming at stabilizing the “financial capital” of the public, human capital (particularly personal resilience) and social capital (particularly family resilience) are relatively neglected. Third, while “general” social policies may help “averaged” individuals and families, there is a need to take pre-existing family vulnerabilities (such as poverty and caregiving burdens) and inequalities into account when formulating “down to earth” social policies. Fourth, while social science knowledge and theories have important potential contributions to help develop relevant policies and services to promote quality of life under COVID-19, explicit utilization is not strong. With reference to these missing links, we proposed several research and practice directions for the promotion of quality of life under the pandemic.

## COVID-19 and the Psychosocial Well-Being Tsunami

Inevitably, COVID-19 is an unprecedented stressor for individuals and families, triggering realistic or symbolic health threats (Kachanoff et al., 2021), economic insecurity (Shek et al., 2022b), childcare burdens, confinement-related spatial pressures, and disruption of family customs and beliefs in the family system (Feinberg et al., 2022; Prime et al., 2020). Although the COVID-19 epidemic has passed the initial "alert" phase (Shek et al., 2022a) and has entered the “post-resistance" and "recovery" phases, individual and family well-being under the pandemic is still an important issue to be addressed (Morrison et al., 2022; Shek, 2021).

Studies showed that COVID-19 has negative impacts on the psychological well-being of both parents and their adolescent children, such as increased anxiety, depression or distress (Christner et al., 2021; Morelli et al., 2020; Shek et al., 2022b). For children and adolescents, academic stress, spatial resources competition, and less peer support intensified mental health problems. For example, studies in different Chinese contexts showed that the prevalence rates of anxiety (Shek et al., 2022a), depression (Shek et al., 2022b) and post-traumatic stress disorder (Shek et al., 2021b) were disturbing under the pandemic. For parents, financial burdens, extra parenting pressures (Morelli et al., 2020), and work-life balance difficulties (C. Fong & Iarocci, 2020) are precursors of mental health issues. Brown et al. (2020) found that American parents reported experiencing on average about 3.5 stressors, with 68.9% reported experiencing high depressive symptoms and 80.9% experiencing high anxiety symptoms. Christner et al. (2021) showed that 31% of German parents fully agreed they were more stressed than usual. Gadermann et al. (2021) reported that around 44.3% of Canadian parents with non- adult children reported deterioration of psychological well-being during the pandemic.

Besides physical and psychological well-being, research studies also showed that COVID-19 adversely affects dyadic and systemic family processes. With abrupt external uncertainty and stress, the strength of intra-family ties between spouses, parents and children, and siblings is impaired, such as reduction of intimate interactions with fear of infection risk, prolonged cohabitation due to working from home, and removal of social contacts result from isolation. Based on an international sample involving 67 countries, Vigl et al. (2022) found that partnership satisfaction declined at the start of the epidemic compared to participants’ retrospective rating of it prior to this crisis. Genç et al. (2021) also demonstrated that relationship satisfaction indeed was negatively impacted by COVID-19 distress among Turkish Couples. Hsu and Henke (2021) revealed that forced stay-at-home resulting from COVID-19 increased intimate partner violence in roughly three months. The decline in marital and births in the midst of the pandemic in many countries was also reported (Ghaznavi et al., 2022).

Studies also showed deteriorating parent–child relationships in the context of escalating parenting stress, abuse and violence towards the child under the pandemic. Brown et al. (2020) reported that a higher risk of child maltreatment was significantly correlated with greater anxiety and depressive symptom levels in parents. Chung et al. (2020) also reported that parents who were more affected by the financial, resource, and psychological impacts of COVID-19 experienced more parenting stress, which was positively correlated with harsh parenting practices. According to Wang et al. (2021), unemployment under the pandemic in the United States demonstrated increased parent–child conflict, which goes on to predict negative affection in children. Besides, sibling interaction was also disrupted by the pandemic (Toseeb, 2022) such as increased fights due to competition for personal space (Eales et al., 2021). Interestingly, there are also studies showing that the pandemic created time opportunities for 65.4% of parents (Thomson et al., 2021) and closeness opportunities for 49.7% of parents (Gadermann et al., 2021), resulting in increased interactions with children.

Furthermore, there was a “spillover effect” (Ho et al., 2022; Hussong et al., 2022) of individual psychological well-being to dyadic and systemic family well-being. Fosco et al. (2022) reported that while family conflict slightly decreased during the COVID-19 pandemic, harsh discipline increased slightly. As per the children’s report, Hussong et al. (2022) showed a decrease in either parental supportiveness for adolescents, overt family communication, or family satisfaction over the course of the pandemic compared to three years previously. However, Eales et al. (2021) also demonstrated that while 10.4% of parents reported worsened family relationships, 44.5% reported improved relationships. In sum, Bülow et al. (2021) suggested that families significantly differed in the change of family functioning during the lockdown, with relationships improving for some families and deteriorating for others.

## Social Policies in Response to the Pandemic

As the pandemic poses significant public health risks (particularly psychological well- being tsunami) and economic recession to the global community which permeate to impaired quality of life for families (Masarik & Conger, 2017; McCubbin & Patterson, 1983), policymakers had to react fast to mitigate such negative outcomes. Hence, social policies responding to COVID-19 have been devised, including stepping up health care, providing employment and unemployment protection, supplying necessary social assistance, and maintaining social stability.

To safeguard physical well-being and to avoid the risk of greater health system malfunctions or even collapse (Forman & Kohler, 2020), both developed and developing societies have been struggling with providing sufficient coverage of testing, treatment and care with respect to COVID-19. In Germany, the German Bundestag approved the “COVID-19 Hospital Relief Act” to mitigate the financial burdens of hospitals and other healthcare facilities (Germany Federal Ministry of Health, 2020). Australia benefits from universal health care established in 1975, supplemented by a private health care system, so that all citizens can be tested and treated for coronavirus, and access to continuing comprehensive primary care via telehealth is a major emphasis of Australia's pandemic response (Shadmi et al., 2020). In mainland China, besides a comprehensive social security system developed prior to the pandemic, medical social insurance, which covers almost all people, was enhanced by including drugs and medical services for the treatment of the new coronavirus as part of the payment arranged for the medical insurance fund (Xinhua, 2020). Unfortunately, we also witness weak and incoherent social policies in low- and middle-income countries (Shadmi et al., 2020) and there is a common concern for worldwide policymakers to strive for fair and equitable access to the vaccine (WHO, 2022).

Besides health policies, much policy attention has been devoted to promote financial security and minimize financial insecurity. Primarily, the most widely used policy surrounds unemployment benefits and employment protection. In EU welfare states, where social security has long been utilized as a robust tool for protecting individuals and families, responded to the COVID-19 crisis by enhancing or developing new income support packages for sick or quarantined workers, the unemployed, and their families, assisting firms in adjusting working hours and preserving jobs (Cantillon et al., 2021). For example, in the Netherlands, the “Emergency Measure for the Preservation of Jobs” (NOW) and “Temporary Emergency Measure for Self-employed Persons” (Tozo) were initiated and existed for almost 18 months (Government of the Netherlands, 2021). Germany has expanded pre-existing policies and provided easier access, such as suspending the wealth test to make vulnerable self-employed people also eligible for the “Hartz IV” unemployment benefits (Cantillon et al., 2021). In Asia, Hong Kong set a special “100% Personal Loan Guarantee Scheme” (PLGS) provided an extra financing option for the unemployed, and the “2022 Employment Support Scheme” has been launched (HKSAR Government, 2022). Singapore introduced several financial protection schemes, including the “COVID-19 Support Grant”, “Jobs Support Scheme” and “Jobs Growth Incentive” (Gentilini et al., 2022). In North America, Both Canada and the United States have typically been categorized as liberal welfare state polities with roughly the same and relatively low levels of social spending, although Canada distinguishes itself as relying extensively on and enhancing the universal social safety net represented by federal Employment Insurance (Béland et al., 2021), and additionally introducing two large programs of “Canadian Emergency Response Benefit” and “Canada Emergency Wage Subsidy”. By comparison, the United States turned to a series of large stimulus bills and Federal Reserve actions, including “Families First Coronavirus Response Act” help to address paid sick leave and unemployment benefits (Gentilini et al., 2022).

Besides employment and unemployment policies under COVID-19, different governments also proposed cash transfers or voucher schemes. The United Kingdom government raised the benefit of its main state-paid social protection program (the Universal Credit, UC) and designed at speed a large emergency package (Hick & Murphy, 2021), such as launching a one-off cash transfer of £500 for those working households that receive tax credits and providing the “COVID Local Support Grant” to support families with bills for food, key utilities, and other essentials (Gentilini et al., 2022). In Singapore, four stimulus packages were launched, including the “Unity Budget”, the “Resilience Budget”, the “Solidarity Budget”, and the “Fortitude Budget” by providing families with the one-off grant, additional cash payout, top-up, vouchers, and electronic devices (Singapore Economic Development Board, 2020). Furthermore, there are some initiatives for supporting work-family balance. Germany and Belgium extended the pre-existing system of insurance-based parenthood leave (Cantillon et al., 2021). As part of the “Second Novel Coronavirus Disease (COVID-19) Emergency Response Package”, the Japanese government announced that self-employed parents would be entitled to get a daily subsidy when they have to take care of their kids and are unable to work due to a scarcity of childcare facilities or school closures (Gentilini et al., 2022).

The development of COVID-19 policies in early 2020 was constrained by the surprise occurrence and uncertainty surrounding the pandemic. After more than 32 months, we observe that there are several gaps in social policies in response to COVID-19 which would undermine the quality of life of the general public. First, in contrast to physical well-being policies on COVID-19 on vaccination, infectious disease control and treatment, there are comparatively fewer policies on mental health or psychological well-being. As COVID-19 can cause severe physical impairment and even death, it is reasonable to place a strong emphasis on physical health.

However, it should also be noted that negative emotions such as fear, anxiety and depression associated with COVID-19 are not uncommon (Yao & Wu, 2022). As “no health without mental health” (Prince et al., 2007), COVID-19 health policies without reference to psychological well- being are obviously deficient. From a holistic psychological well-being perspective, besides the reduction of negative psychological well-being, there is also a need to promote positive psychological well-being, such as thriving and life purpose under COVID-19. Essentially, how can one grow positively under COVID-19? When we face so many COVID-19 related deaths, how can we find life meaning? There are studies showing that policy stringency was positively related to poor mental health (Aknin et al., 2022). Moreno et al. (2020) outlined a position paper on mental health challenges under COVID-19 and suggested that the pandemic could help to improve the mental health system. There are also views that suggest the significance of keeping a keen eye on the mental health of the general public under the pandemic (Goldman et al., 2020; Pfefferbaum & North, 2020). Unfortunately, it seems that this is an unrealized ideal. In a review of the policies responding to COVID-19, it is remarked that “other important health issues are not mentioned at all in the sample of 67 evaluations … for example the impact of the pandemic on mental health” (OECD, 2022, p. 7). Villarreal-Zegarra et al. (2022) also showed that “there is limited evidence available to evaluate national and local policies aimed at directly or indirectly preventing or ameliorating mental health problems at work during the COVID-19 pandemic” (p. 2).

The second observation is that most social policies focus on “financial capital”, such as employment protection and unemployment benefit (OECD, 2022). Undeniably, financial protection under the pandemic is fundamental because it helps to contain the rapid deterioration of quality of life (e.g., Ikeda et al., 2022). However, while financial security is important, we have to ask whether financial capital alone can solve all quality of life issues arising from the pandemic. From a well-being perspective, money may contribute to hedonic well-being (such as life satisfaction) but not eudaimonic well-being (such as finding life meaning). In the same vein, financial security may help to reduce family conflicts but not promotion of family cohesion under the pandemic. In short, we have asked besides financial capital, what other forms of capital should be fostered under the pandemic. Our argument is that while money is important, financial capital alone is not enough. In fact, two other forms of capital, including human capital and social capital, are important.

Human capital refers to the knowledge and competencies of an individual (Garavan et al., 2001). Obviously, knowledge about COVID-19 preventative behaviors is important under COVID-19. However, knowledge and skills on COVID-19 prevention are not the whole story. Some other skills are important, such as emotional management, resilience and living a meaningful life under the chaos of the pandemic. In fact, there are studies showing that developmental assets are positively related to psychological well-being under the pandemic. One example is personal resilience.

There are three lines of studies regarding the importance of personal resilience under the pandemic. First, there are studies showing that around one-third of the participants in Italy failed to achieve the resilience criteria (Panzeri et al., 2021) or showed low levels of resilience in a Polish sample (Skalski et al., 2022). However, based on longitudinal data collected in Australia, To et al. (2022) demonstrated that individual resilience values did not significantly alter over the COVID-19 outbreak in Australia. Second, researchers reported a positive correlation between personal resilience and one's psychological well-being: To et al. (2022) reported resilience was negatively associated with psychological distress; Li et al. (2021b) reported negative relationships between resilience and negative emotions in Chinese college students; Wister et al. (2022) showed that elderly adults who possessed greater multimorbidity resilience were less prone to worry about the virus and perceived less negative effects of such a health crisis; Karataş and Tagay (2021) showed that resilience was significantly and positively associated with hope, meaning in life, and life satisfaction among Turkish adults. Besides, research supported that resilience serves as a significantly strong predictor of quality of life, involving physical health, mental health, social bonding, and environmental variables (Keener et al., 2021). Skalski et al. (2022) further provided evidence that persistent thinking about COVID-19 may operate as a mediator in the link between personal resilience and individual well-being. Meanwhile, Li et al. (2021a) showed that resilience was positively associated with mental health. Third, there are also studies showing that individual resilience mediated between COVID-19-related threats and personal mental health (Labrague & de los Santos, 2021; Yıldırım & Solmaz, 2022) and individual resilience was a protective factor (Guillasper et al., 2021). Resilience, according to Havnen et al. (2020), not only buffered the direct effect of stress on anxiety, but also moderated the indirect impact of stress on depressive symptoms under the pandemic.

Besides “human capital” in terms of personal resilience, social capital is also important. In fact, studies demonstrate that social capital was positively associated with adjustment under the pandemic (Bartscher et al., 2021; Pitas & Ehmer, 2020). Ironically, with the policy of maintaining “social distancing”, virtual social networks can mainly be formed only via the internet. Besides, although governments form panels of expert advisors, they are dominated by medical practitioners with very little involvement of NGOs and civic communities.

With social distancing, home is the safest place for people under the pandemic. Besides, as “illness is a family affair” (Wright & Bell, 2009, p. ix), there is a need to understand how family social capital, such as family resilience, is related to the quality of life under the pandemic (Wang et al., 2022). Theoretically, there are theories highlighting the importance of family resilience in facing family crises, such as the stressors arising from the pandemic. For example, the family resilience framework raised by Walsh (1996, 1998) provides a good conceptual tool for researchers and practitioners to promote family well-being under the pandemic. The family resilience framework determines and integrates important family processes from three areas of family functioning, including “belief systems”, “organizational patterns”, and “communication processes”, with three domains in these areas (i.e., nine domains in total), namely, “making meaning of adversity, positive outlook, and transcendence and spirituality”, “flexibility, connectedness, social and economic resources”, and “clarity, open emotional expression, and collaborative problem solving” that correspond to each area respectively (Walsh, 2002, p. 132). Instead of simply looking at the specific attributes of family members individually, the concept of family resilience focuses on interactive processes, it focuses on “processes that foster relational resilience as a functional unit” (Walsh, 1996, p. 261).

Empirically, studies showed that at least one-tenth of the families showed low family resilience under the pandemic (Eales et al., 2021; Family Action, 2021). Besides, studies revealed a negative relationship between family resilience and family well-being: Ramadhana (2020) showed that family resilience was positively correlated with happiness, satisfaction and relief, while negatively associated with negative emotions during the epidemic; Ho et al. (2022) reported a significant association between family resilience and lower COVID-19 psychological impact after controlling for the risk factor of financial hardship; Zhuo et al. (2022) demonstrated the positive predictive value of family resilience in relation to adolescent mental health. There are also a few studies showing the protective role of family resilience on well-being in the COVID-19 pandemic context (Giordano et al., 2023; Zhang et al., 2022). For example, Chan et al. (2021) reported that the negative relationship between COVID-19-related stressors and depression among Minnesota participants could be buffered by family problem-solving capacity and efficient communication, and a positive family prospect significantly moderated the association between these stressors and anxiety in Hong Kong participants.

Given the overwhelming evidence that family resilience is negatively correlated with psychological morbidities, Gayatri and Irawaty (2022) suggested the importance of promoting family resilience as a flexible way to collaboratively cope with the epidemic crisis, including creating daily gratitude practices, promoting family communication, fostering shared positive mindsets, and facilitating social support. Unfortunately, current studies have mostly concentrated on the performance of individual protective factors in response to the crisis, and less on the protective role and contribution of the nearest social-ecological system that encircles individuals, i.e., the family (Ho et al., 2022).

The third observation is that the needs of vulnerable groups and social inequalities are not adequately addressed under the “generic” COVID-19 policies. Chen et al. (2022) pointed out that “family income level and race/ethnicity play a significant role in the lives of families coping with a variety of challenges due to the pandemic” (p. 719). Andrade et al. (2022) similarly concluded that the pandemic has hit families experiencing economic disadvantage, ethnic minority families, vulnerable groups and women harder. Unfortunately, as revealed by the evaluation study by OECD (2022), “issues relating to policies’ proportionality and coherence are still largely underexplored – at the same time, they may be particularly useful for policy debate when resources are scarce and cross-government co-ordination is crucial” (p. 35).

With reference to intergenerational relationships and family caregiving policy, Stokes and Patterson (2020) pointed out that “sandwich” generation adults, ethnic minorities and people with lower socio-economic attributes require specific policies targeting their challenges and problems faced under the COVID-19 pandemic focusing on flexibilities. They also emphasized that COVID-19 could be transmitted via families and intergenerational relationships where women are usually the caregivers of the old and young family members. Hence, they suggest that diversity of family structure, as well as family caregiving, should be considered in promoting balanced work and caregiving responsibilities. Phillips et al. (2020) also argued that challenges and additional responsibilities are shouldered by “unpaid family cares” (p. 1). Highlighting the “forgotten” care economy (core economy, reproductive economy or hypocrisy economy), Power (2020) pointed out that women shouldered the main bulk of “unpaid care work” before the pandemic and such unpaid work had increased substantially after the onset of COVID-19. Zanoni (2021) similarly warned that “the disruption of capitalist flows by the pandemic has exacerbated the cleavages and power inequalities” (p. 580).

One policy implication of these criticisms is that while the “generic” policy (such as employment protection) is definitely helpful, it is not enough for families experiencing vulnerability such as having family members with mental health problems. Burgess (2020) pointed out that “diagnosis is rarely a solution to problems faced by poverty and inequality” and “political- economy of mental health” definitely matters (p. 1) so that anxiety-management applications would not be sufficient for systemic vulnerabilities. Cooney (2020) also remarked that the failure to consider caretaking and family burdens as systemic problems is showing the “farce of our societal approach to separating work and family lives”. In the same vein, Daly (2022) remarked that “a basic problem is that we have not devised an equality respecting system to replace the full- time caretaking labour of women in the home” (p. 7). C. Fong and Iarocci (2020) also argued for implementing inclusive and flexible “family-friendly” policies such as universal paid sick leave as well as financial assistance for parents of front-line employees who face a greater risk of getting infected (p. 1124). Based on such views, policies incorporating progressive, anti-oppressive, critical and social justice elements should be seriously considered. As argued by Monaghan (2020), we have developed responsive COVID-19 social policies through the lens of a “fractured society” with “class-generated fissures and tensions” (p. 1982).

## Quality of Life Knowledge and Theories

To devise appropriate services and policies to promote quality of life under the pandemic, there is a need to utilize social science knowledge. For example, Bavel et al. (2020) argued that policy-makers should better utilize social science knowledge to develop appropriate responses to the pandemic. These include stimulation of a shared vision of purpose and joint sense of identity, identification of credible sources to promote public health messages, promotion of cooperative behavior and prosocial behavior, provision of public health information to marginalized communities, focus on self-benefit and other benefits of preventative behavior, differentiation of misinformation, disinformation and correct information, as well as utilization of “physical distancing” instead of “social distancing” (which means cutting off social ties). Unfortunately, it seems governments have not seriously considered these suggestions in formulating COVID-19 policies in reality.

As far as theoretical models on the impact of COVID-19 on individual and family well- being, many social science theoretical models can be considered. For example, researchers can borrow the Family Stress Model (FSM) to understand the relationships amongst economic hardship, stress, parental distress, parenting, and child adjustment. The basic thesis of this model is that economic pressure causes parents emotional distress and inter-parental conflict leading to disrupted parenting, which would directly affect child and adolescent adjustment problems (Masarik & Conger, 2017). Although the FSM primarily focuses on economic stress, it has been applied to various environmental stressors (Masarik & Conger, 2017), including COVID-19 (Lee et al., 2022; Lucassen et al., 2021).

Besides, researchers can utilize the ABCX Model (Hill, 1958) to understand family adaptation under the pandemic. The ABCX Model asserts that the hardship of stressors, accompanying the family's definition of it, along with the resources available, determines the extent to which the stressor transformed into a crisis. Based on the ABCX model, McCubbin and Patterson (1983) expanded the theory to include three components (pre-crisis, crisis, and post- crisis) and five post-crisis variables, such as “perception of the initial stressor”, “pile up”, and “existing and new resources”. Based on longitudinal data, Adesogan et al. (2022) discovered that pre-pandemic stressors (e.g., financial pressure) were closely correlated with psychological health over the duration of the pandemic, which was emphasized in the double ABCX model. Tokatly Latzer et al. (2021) also revealed that negative attitudes amongst parents under COVID-19 lockdown were associated with deterioration or regression in children’s behavior.

Linking COVID-19-induced social disruption to child adjustment, a conceptual framework involving the wellbeing of caregiver and holistic family functioning was formulated by Prime et al. (2020) mainly based on family systems theory (Bowen, 1974), family stress model (Conger & Conger, 2002), and Walsh’s (1998) family resilience framework. This framework started with the “social disruption” generated by the pandemic, which could affect children’s adjustment not only directly but also through the mediating variable of “caregiver well-being”. In addition, the model emphasizes multilevel ecological organization, assuming that the role of family structure, including individuals, dyadic system (i.e., marital, parent-child, sibling subsystems) and the whole family, should be considered in the association between “caregiver well-being” and “child adjustment” (Prime et al., 2020). Furthermore, the model proposes that “pre-existing family vulnerabilities,” such as economic hardship, special needs, or “preexisting strengths,” such as family relationships, would exacerbate or buffer the foregoing processes.

Regarding theories to be utilized under COVID-19, we should note three points. First, it is desirable to adopt an ecological model integrating micro and macro factors, particularly family ecological factors focusing on family resilience. Second, pre-existing family vulnerabilities such as poverty and gender inequality must be taken into account so that social policies with a “human face” could be devised. Finally, social policies should be considered using the lens of progressive, anti-oppressive and social justice perspectives. Essentially, as Monaghan (2020) remarked, we have to ask “what sort of society are we heading towards and what sort of world do we want to share?” (p. 1982).

## Research and Practice Direction

With reference to quality of life under the pandemic, there are several research directions we could consider. First, as COVID-19 is ongoing and the aftermaths of the pandemic last over time, there is a need to conduct longitudinal studies which can capture the dynamic changes associated with the pandemic. In particular, it is important to understand how “Long COVID” would affect oneself, the family and the community over time. Second, how COVID-19 affect developmental outcomes may be mediated and/or moderated by other factors, such as socio- demographic factors and psychosocial processes. Third, it is theoretically important to ask whether the “general” stress-coping models are applicable under the pandemic context. For example, with reference to the integrated model proposed by Prime et al. (2020), there are many possibilities for testing the different pathways within the model. Fourth, with reference to the argument that human capital (indexed by individual resilience) and family social capital (indexed by family resilience) are also important capital besides financial capital, the possible relationships between individual resilience and family resilience should be further explored. Fifth, we need more research on how the resilience processes differ across Western and non-Western contexts, such as family communication. Sixth, mixed-method research including quantitative and qualitative strategies would be necessary to understand quality of life under the pandemic. Finally, more evaluation studies of social policies responding to COVID-19 are necessary.

There are several practice directions as far as the promotion of quality of life is concerned. First, we have to reiterate the importance of maintaining the quality of life under the pandemic. Hence, it’s critical to recognize and pinpoint those who might be “at-risk” of psychological well-being at an early stage. Second, it is important to promote the significance of individual quality of life and family quality of life. In particular, how individual resilience and family resilience might contribute to the quality of life should be seriously considered. Hence, besides having messages such as “vaccinate for oneself and others” and “maintaining personal hygiene under the pandemic”, messages such as “have positive family energy under the pandemic” and “build up family cohesion under COVID-19” are also important messages for the public.

Third, it is important to cultivate personal resilience under the pandemic. Besides programs to manage negative emotions and reduce psychological morbidity, it is vital to strengthen factors that contribute to personal resilience, such as finding positive meaning, getting more social support, and healthy management of one’s emotions. Fourth, it is vital to boost families’ quality of life via the promotion of family resilience. With lockdown and social distancing measures, the promotion of family communication and cohesion is important. Besides, in urban settings, how to promote neighborhood support is also another important intervention angle. With community lockdown, neighborhood support plays an important role in supporting high-risk families. In Hong Kong, in collaborating with four non-governmental organizations, we are now implementing a family resilience project aiming to promote family resilience in families in Hong Kong (https://family-fhss.polyu.edu.hk). Faced with family financial capital problem, human capital (personal resilience) and family social capital (family resilience) are definitely helpful to deal with family problems arising from the pandemic.

Fifth, there is a need to address the specific and unique needs of groups with “pre-existing” vulnerabilities such as families with members suffering from COVID-19, caregiving burdens, and economic disadvantages. Hence, although “uniform and general” programs focusing on financial capital are important, they may not be enough to address the needs of the vulnerable groups. Sixth, we need good scientific theories to support policies to promote families under COVID-19. Obviously, the integrated theory of Prime et al. (2020) is an excellent starting point. As we argue above, we may consider adding human capital (individual resilience) and macro policies (regular and COVID-19 specific ones) to this model.

Finally, while many “ad hoc” social policies are devised in response to COVID-19, it is necessary to think about how to strengthen family resilience in a situation without COVID-19 (i.e., “regular” social policies). For example, one should have regular exercise to prevent heart attack. It may be too late to have regular exercise when one already has had a heart attack. In the same vein, we should promote personal resilience and family resilience in the days without COVID-19 through systematic programs. For example, there is a need to promote life skills in young people and there is in fact a strong demand for such “soft skills education” (Shek et al., 2021a). As highlighted in OECD (2022), “pandemic preparedness was generally insufficient, particularly in light of the major human and financial costs associated with global health crises similar to the COVID-19 pandemic” (p. 2).
